# Convenient and effective ICGylation of magnetic nanoparticles for biomedical applications

**DOI:** 10.1038/s41598-017-09627-x

**Published:** 2017-08-18

**Authors:** Hye Sun Park, Jongwoo Kim, Mi Young Cho, Hyunseung Lee, Sang Hwan Nam, Yung Doug Suh, Kwan Soo Hong

**Affiliations:** 1 Bioimaging Research Team, Korea Basic Science Institute, Cheongju, 28119 Korea; 2 Immunotherapy Convergence Research Center, Korea Research Institute of Bioscience and Biotechnology, Daejeon, 34141 Korea; 3 Laboratory for Advanced Molecular Probing (LAMP), Research Center for Convergence NanoRaman Technology, Korea Research Institute of Chemical Technology, Daejeon, 34114 Korea; 4 School of Chemical Engineering, Sungkyunkwan University, Suwon, 16419 Korea; 5 Graduate School of Analytical Science and Technology, Chungnam National University, Daejeon, 34134 Korea

## Abstract

Nanoprobes used for biomedical applications usually require surface modifications with amphiphilic surfactants or inorganic coating materials to enhance their biocompatibility. We proposed a facile synthetic approach for the phase transfer of hydrophobic magnetic nanoparticles by the direct adherence of fluorescent probes, without any chemical modifications, for use as a magnetic resonance (MR)/near-infrared (NIR) fluorescence bimodal imaging contrast agent. Indocyanine green (ICG) was used not only as an optical component for NIR imaging, but also as a surfactant for phase transfer with no superfluous moiety: we therefore called the process “ICGylation”. Cell labeling and tracking *in vivo* with ICGylated magnetic nanoparticles were successfully performed by MR/NIR dual-mode imaging for three days, which showed remarkable biostability without any additional surface functionalization. We expect that this novel MR/NIR contrast agent demonstrating sensitive detection and simultaneous imaging capability can be used in diverse fields, such as the imaging and tracking of immune cells to confirm immunotherapeutic efficacy. The approach used could also be applied to other kinds of nanoparticles, and it would promote the development of advanced functional multimodal nanobioprobes.

## Introduction

Magnetic nanoparticles (MNPs) have been used in various fields^1^ such as those involving magnetic resonance imaging (MRI)^2^, biomedicine^3^, catalysts^4^, and data storage^5^. In particular, there has been an increasing interest in cell labeling and tracking based on MRI because of its noninvasive nature and the high resolution it offers^6–9^. Immune cells labeled with MNPs have been recently used for *in vivo* tracking for immune cell-based therapies as well as immunological research^6^.

For biomedical applications, additional surface modification of the MNPs is usually necessary because of their instability owing to their high surface-to-volume ratio and chemical activity^10–13^. Amphiphilic surfactants or inorganic molecules such as lipid-poly(ethylene glycol)^14–16^, copolymers based on dextran derivatives^17^, or silica shells^18, 19^ have been introduced as surface-coating materials for stabilization and functionalization in biological environments^20–22^.

Moreover, there has been an increased demand for multimodal imaging using combinations of modalities to provide complementary and diverse information, such as MR/optical^23–25^, MR/PET (positron emission tomography)^26^, and MR/PET/optical^27^ techniques. For optical imaging, near-infrared (NIR) fluorescence is used widely; it enables non-invasive *in vivo* imaging as well as *ex vivo* histological analyses with sensitive detection^28, 29^. Accordingly, the combination of MR and NIR fluorescence imaging has undergone intensive investigation^30^. In particular, indocyanine green (ICG), a NIR fluorescent probe approved by the U.S. Food and Drug Administration, has already been used in clinical applications^31–36^. However, its applications are still limited owing to the difficulties of combining it with other imaging components, and long-term *in vivo* tracking of labeled cells with ICG is challenging owing to its non-functional moiety and poor stability^31^. No approach has been proposed in which ICG is used both as an optical probe and as a stabilizer of nanoparticles.

**Figure 1:**
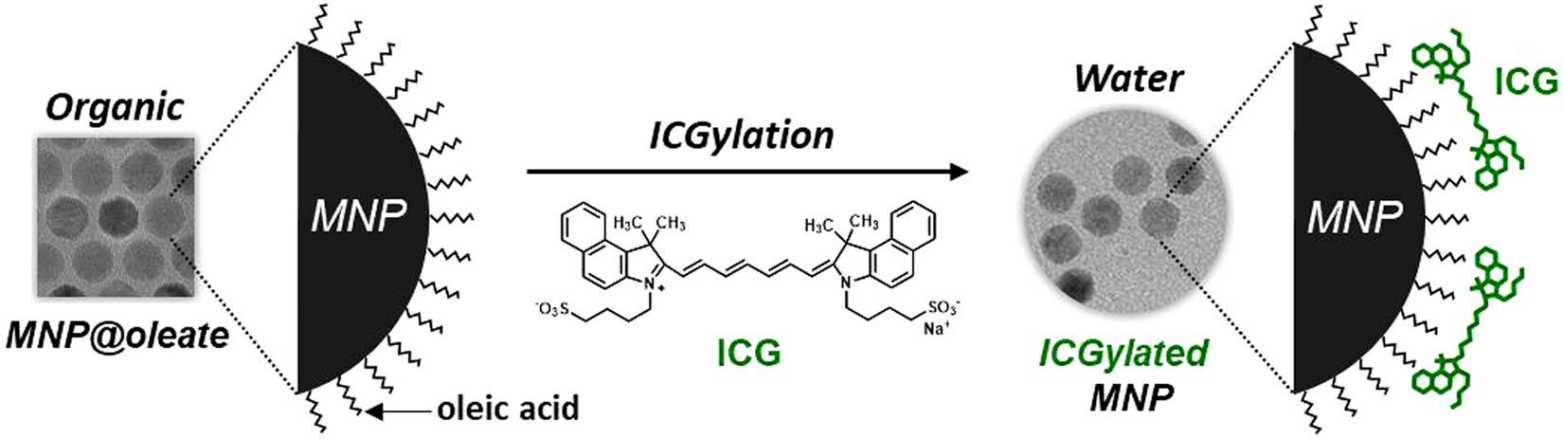
Schematic illustration of preparation of ICGylated MNPs. Hydrophobic magnetic nanoparticles (MNPs) were coated with indocyanine green (ICG) molecules, which enable their phase transfer to aqueous media.

Herein, we used ICG not only as an optical component for NIR imaging, but also as a surfactant for phase transfer with no superfluous moiety of MNPs — we call the technique “ICGylation”. ICGylated MNPs were synthesized by a novel and facile approach for the phase transfer of hydrophobic MNPs by direct adherence of ICG. We demonstrated that several immune cell lines were successfully labeled with ICGylated MNPs and that the labeled cells can be used for tracking in a mouse model for three days, as illustrated in Fig. 1.

## Results and Discussion

### Synthesis and characterization of ICGylated MNPs

**Figure 2:**
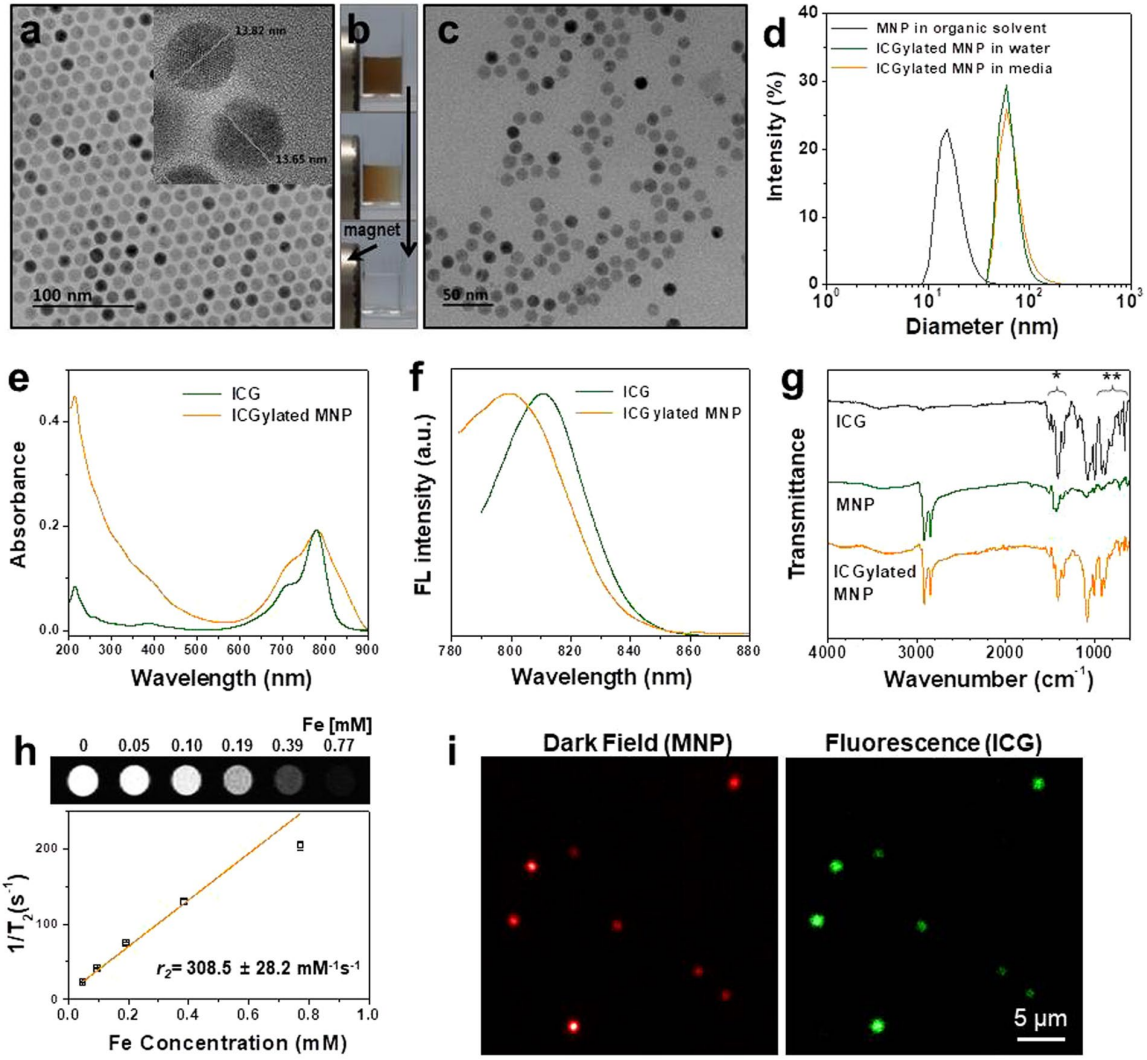
Characterization of ICGylated MNPs. (**a**) Transmission electron microscopy (TEM) image of MNPs in an organic solvent. (**b**) Magnetic separation procedure of ICGylated MNPs. (**c**) TEM image of ICGylated MNPs in water. (**d**) Size distribution of MNPs in an organic solvent and ICGylated MNPs in aqueous solvents. (**e**) Absorption and (**f**) fluorescence (emission scan at λ_ex_ = 765 nm) spectra of ICG and ICGylated MNPs. (**g**) FT-IR spectra of MNPs, ICG, and ICGylated MNPs (^*^aromatic C = C stretches, ^**^C-H out of plane bending). (**h**) T_2_ relaxation rate (bottom, 1/*T*_2_, s^−1^) and T_2_-weighted MR images (top) as a function of Fe concentration for ICGylated MNPs. (**i**) Dark-field (left) and fluorescence microscopy (right) images of ICGylated MNPs at a single-particle level. MNPs are shown in the dark-field image; ICG is shown in the fluorescence image.

MNPs were synthesized by a two-step thermal decomposition procedure^37^ and were observed by high-resolution transmission electron microscopy (HRTEM) images (Fig. 2a) to exhibit a uniform size distribution with a diameter of around 13.7 nm. ICG was coated onto the surface of MNPs using a single emulsion evaporation method wherein ICG molecules were dissolved in water and MNPs were dispersed in an organic phase: we termed this process ICGylation of MNPs. To remove uncoated free ICG molecules, magnetic separation was performed several times (Fig. 2b). After ICGylation, TEM images (Fig. 2c) showed that the ICGylated MNPs were well dispersed in water and in a cell culture medium. The sizes of the ICGylated MNPs measured by dynamic laser scattering (DLS) were in the range 63.1 ± 15.3 nm in water and 68.4 ± 22.2 nm in the medium, whereas those of MNPs in the organic solvent lay in the range 16.9 ± 4.9 nm (Fig. 2d). The size distributions suggest several layers of ICG molecules on the MNPs without large aggregates. The zeta potential of the dissolved ICG shifted from −25.1 to −41.6 mV after ICGylation (Supplementary Fig. 1a), and this result was not affected by the concentration of ICG or ICGylated MNP solutions.

ICG molecules have two hydrophobic polycyclic groups and two hydrophilic sulfate groups. The hydrophobic part of ICG is easily embedded in the hydrophobic core of proteins^38^. The hydrophobic parts of the ICG molecules were adsorbed on the particle surface via hydrophobic interactions, while the hydrophilic parts were more exposed to the water phase, which enhanced the negative charge of the ICG molecules. We found that ICG molecules act as surfactants for phase transfer owing to their amphiphilic property in a solution of ICGylated MNPs.

After ICGylation, the peak of the absorption spectrum of ICG molecules on MNPs shifted slightly from 778.8 nm to 781.0 nm and broadened (Fig. 2e), and the peak for the emission spectrum shifted from 810.7 nm to 799.6 nm (Fig. 2f). These results are consistent with previous reports indicating that ICG molecules interact with their surroundings^38, 39^. Additionally, as demonstrated by the Fourier transform infrared (FT-IR) spectra of the ICG molecules and ICGylated MNPs (Fig. 2g), the intensities of several peaks related to the motions of the aromatic planes of ICG molecules, aromatic C = C stretches (1400–1500 cm^−1^), and c-h out-of-plane bending (600–1000 cm^−1^) decreased and = C-H vinyl stretches (900–1100 cm^−1^) shifted to higher wavenumbers (Supplementary Fig. 1b)^40^. These observations may suggest restricted motion of ICG molecules embedded in oleic acid-capped particle surfaces. With respect to the magnetic property, the ICGylated MNPs showed a high T_2_ relaxivity (*r*_2_), approximately 308 mM^−1^ s^−1^, as shown in Fig. 2h. Dark-field and fluorescence images of the ICGylated MNPs were obtained at the single particle level (Fig. 2i). The scattering from MNPs and fluorescent signals from ICG molecules were highly co-localized and their intensities corresponded to each other, indicating the direct adhesion of ICG molecules to the particle surfaces. The shift in the zeta potential, the FT-IR spectra of the ICGylated MNPs with respect to those of free ICG molecules, and the co-localization of the dark-field and fluorescence images indicate that the ICG molecules were effectively coated on the MNPs using our proposed method.

### Photostability of ICGylated MNPs

**Figure 3:**
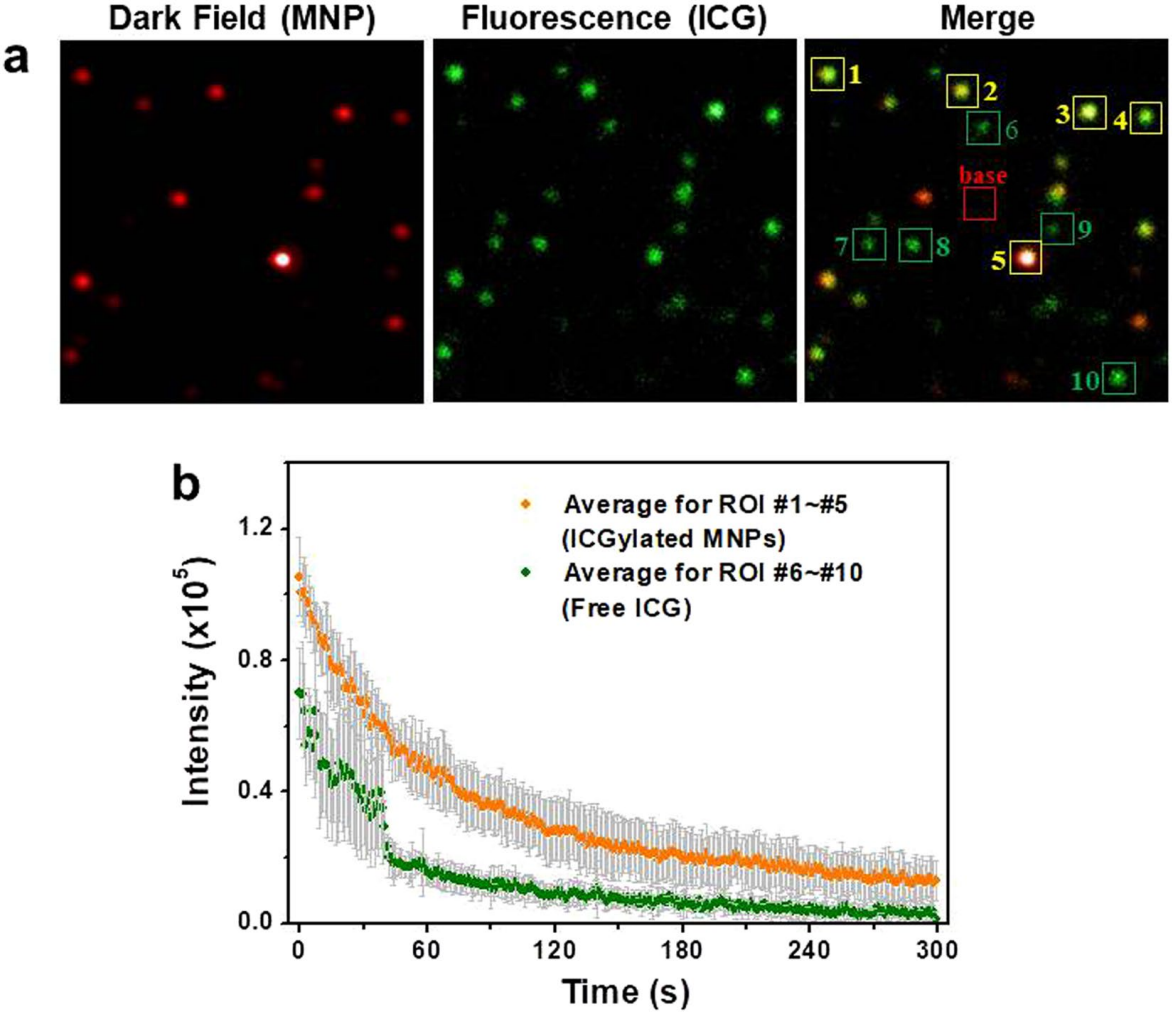
Enhanced photostability of ICG after ICGylation. (**a**) Dark-field (red), fluorescence (green), and merged images of ICGylated MNPs. MNPs are shown in the dark-field image; ICG is shown in the fluorescence image. In the merged image, the ICGylated MNPs appear yellow, whereas the free ICG molecules appear green. Yellow boxes (1–5) and green boxes (6–10) were selected as ROIs to measure the fluorescence signals for coated ICG and free (uncoated) ICG, respectively. (**b**) Averaged fluorescence intensities under continuous radiation (at 785 nm) for each ROI. Individual fluorescence intensities for each ROI are indicated in Supplementary Fig. 2.

To compare the photostability of free ICG molecules and the ICGylated MNPs, we added free ICG molecules to a solution of ICGylated MNPs. Dark-field and fluorescence images of the mixed solution were obtained (Fig. 3a). The ICGylated MNPs were observed in both dark-field and fluorescence images, whereas free ICG molecules were only observed in the fluorescence image. Thus, we could distinguish the ICGylated MNPs from free ICG molecules in the merged images. We analyzed the average fluorescence intensity as a function of the irradiation time in 5 arbitrarily chosen regions of interest (ROIs) for the two cases to compare the decrease rate under continuous irradiation (Fig. 3b). The time-intensity profile for each ROI is shown in Supplementary Fig. 2. We observed a fluctuation in the intensity within 1 min in the case of free ICG molecules, attributed to photoblinking and photobleaching, similar to the usual behavior of organic dyes; however, we could not find any fluctuation in the case of ICGylated MNPs. After irradiation for 5 min, fluorescence signals were rarely detected for free ICG owing to rapid photodegradation, but could be detected for ICGylated MNPs. These results indicate that the photostability of ICGylated MNPs is enhanced by adsorption of ICG molecules on the particle surface. We assumed that the ICG molecules bound to the hydrophobic surface were more stable than the free ones in water, which was consistent with previous reports concerning fluorophores in hydrophobic environments^39, 41, 42^.

### *In vitro* MR/FL imaging of cells labeled with ICGylated MNPs

**Figure 4:**
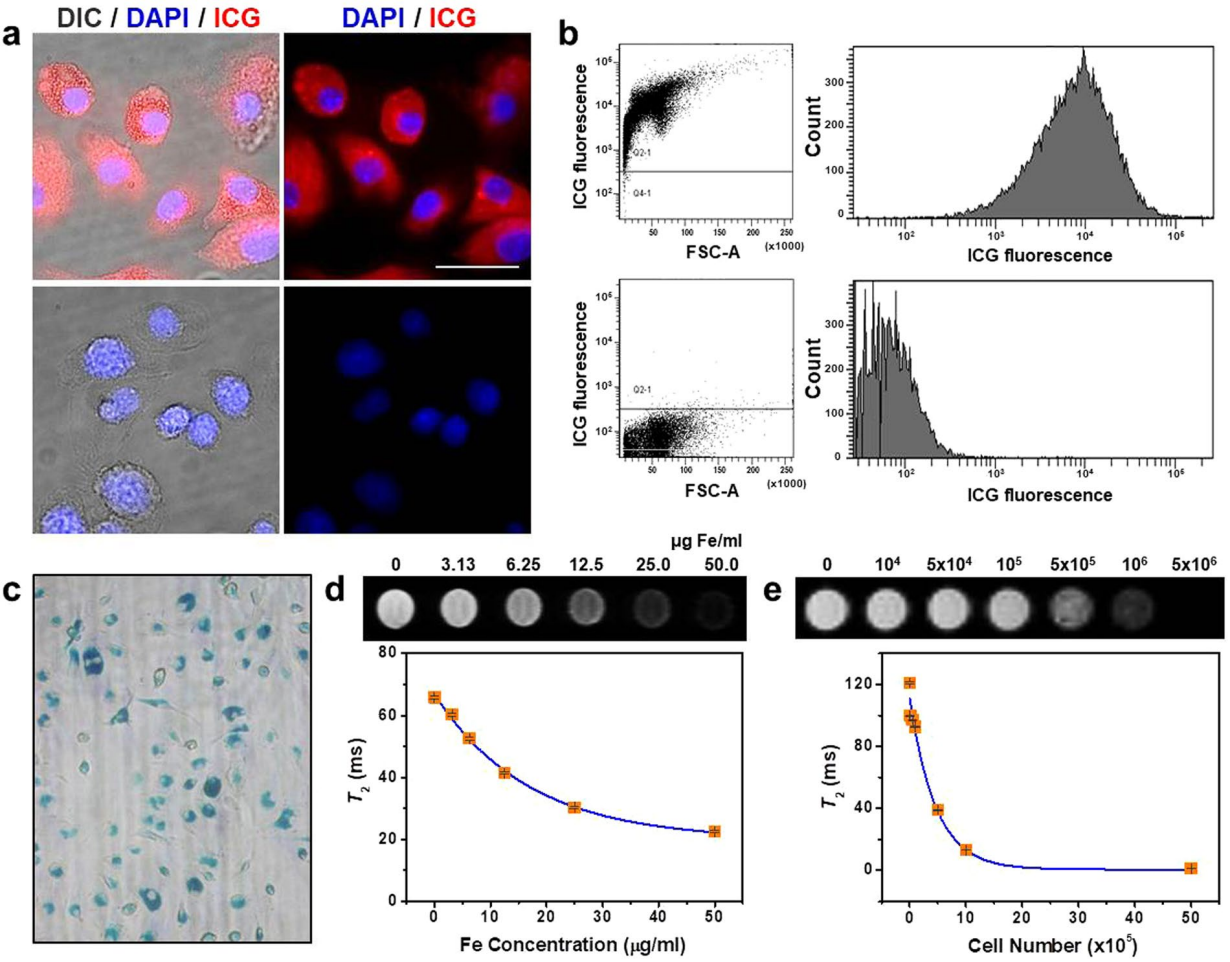
*In vitro* MR/FL imaging of cells labeled with ICGylated MNPs. (**a**) *In vitro* microscope images and (**b**) flow cytometric analysis of the labeled DCs with ICGylated MNPs (top) and those of the unlabeled ones (bottom). Blue and red colors represent DAPI-stained nuclei and ICG fluorescence. The scale bar indicates 15 μm in (**a**). (**c**) Prussian blue staining of the labeled DCs incubated with 25 µg Fe/ml of ICGylated MNPs for 24 h. (**d**) T_2_-weighted MR images (top) and *T*_2_ values (bottom) for the labeled DCs (5 × 10^6^) as a function of treated Fe concentration. (**e**) Same as (**d**) but as a function of the cell number with DCs labeled at a concentration of 100 µg Fe/ml of ICGylated MNPs.

To conduct MR and fluorescence dual-mode imaging using ICGylated MNPs, we first performed intracellular delivery of the particles into bone marrow-derived dendritic cells (BMDCs). *In vitro* cellular fluorescence images (Fig. 4a) and flow cytometric analysis (Fig. 4b) of the DCs incubated with ICGylated MNPs and DCs not incubated were obtained. Fluorescence of ICG molecules coated on the particles was clearly observed for the incubated DCs but not for the control. In Prussian blue (PB)-stained images (Fig. 4c), staining was effectively detected in the cytosol for most cells, indicating that the BMDCs were successfully labeled with the ICGylated MNPs.

To measure the MR imaging sensitivity of the labeled cells, T_2_-weighted MR images and *T*_2_ values were obtained as functions of treated concentrations (Fig. 4d) and labeled cell numbers (Fig. 4e). These factors could affect sensitive detection of the labeled cells following their redistribution among neighboring cells or tissues after *in vivo* administration. The *T*_2_ value of DCs labeled with ICGylated MNPs was only 10% of that of unlabeled DCs for 1 × 10^6^ cells, indicating their excellent MR imaging sensitivity.

We also adopted the same approach for two other cell lines: DC2.4 and RAW264.7 cells (Supplementary Fig. 3); the ICGylated MNPs were also effective in labeling these two cell lines. The cell viability of the three cell lines after labeling with the ICGylated MNPs was also measured as a function of iron concentration (Supplementary Fig. 4), revealing that the cytotoxicity of the ICGylated MNPs in the cell lines was negligible.

The photostability of the cells labeled with ICGylated MNPs was also investigated (Supplementary Fig. 5). NIR fluorescence images of DCs labeled with ICGylated MNPs (Supplementary Fig. 5a) and those labeled with ICG (Supplementary Fig. 5b) were obtained. Cells were incubated with ICGylated MNPs or ICG molecules for 24 h and collected (2 × 10^6^ cells). The DCs labeled with ICGylated MNPs (ICG-MNP-labeled DCs) and those labeled with ICG (ICG-labeled DCs) as a control were incubated at 37 °C in a CO_2_ incubator for 3 days and fluorescence images were obtained on each day. To compare the relative difference between ICG-MNP-labeled DCs and ICG-labeled DCs, initial fluorescence signals were equally adjusted by controlling the treatment concentrations. Signal-to-noise ratios (SNRs) of fluorescence images for ICG-MNP-labeled DCs and ICG-labeled DCs are shown in Supplementary Fig. 5c. After 2 days of incubation, fluorescence signals of the ICG-labeled DCs were scarcely detected, indicating photo- or thermal degradation of ICG. The fluorescence of ICG-MNP-labeled DCs could be detected even after 3 days of incubation, exhibiting a much slower decrease over time^36^; accordingly, the fluorescence of the ICGylated MNPs was deemed more stable than that of ICG molecules in DCs.

### *In vivo* MR/FL imaging of cells labeled with ICGylated MNPs

**Figure 5:**
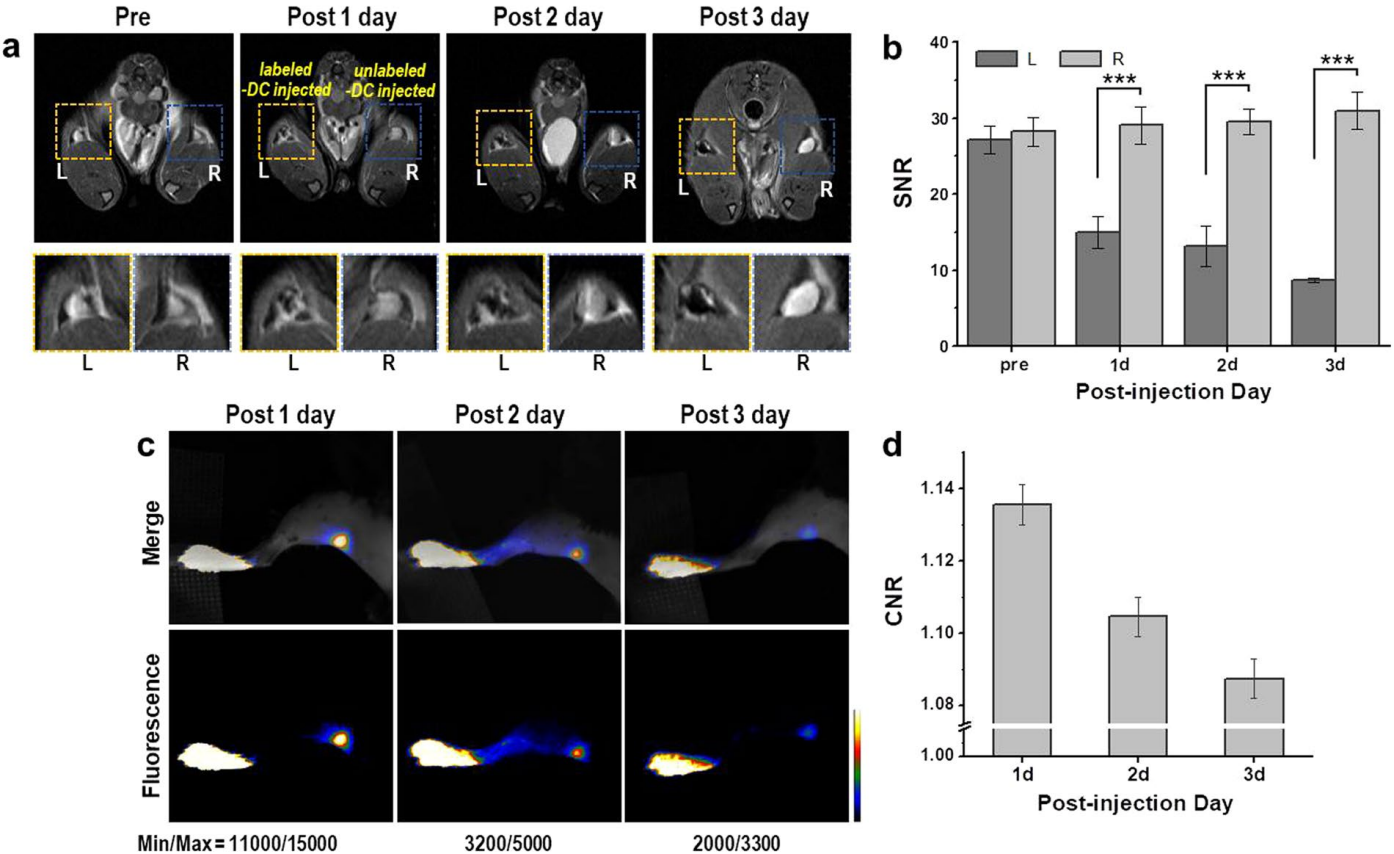
*In vivo* MR/FL imaging of cells labeled with ICGylated MNPs. (**a**) *In vivo* T_2_-weighted MR images of mouse popliteal lymph nodes at different times after injection of DCs labeled with ICGylated MNPs (L, left) and unlabeled (R, right) DCs (2 × 10^6^ cells). (**b**) Signal-to-noise ratios obtained from MR images of (**a**). (**c**) *In vivo* NIR fluorescence images of mouse popliteal lymph nodes after injection of the labeled DCs. (**d**) Contrast-to-noise ratio (CNR) values of the lymph nodes obtained from (**c**). ^***^p < 0.001.

For *in vivo* MR and fluorescence imaging, C57BL/6 mice were subcutaneously injected in the fore- or hind-leg footpads with DCs labeled with ICGylated MNPs or unlabeled ones. After injection with labeled (left pad) and unlabeled (right pad) DCs (2 × 10^6^), MR and fluorescence images of popliteal (Fig. 5) and axillary lymph nodes (Supplementary Fig. 6) were observed for three days. T_2_-weighted MR images of the left popliteal lymph nodes after injection with the labeled DCs (Fig. 5a) showed substantial darkening in the central zone of lymph nodes over time, whereas those of unlabeled DCs did not^7, 43^; a decrease in the signal-to-noise ratio of MR images was also observed for the labeled DCs (Fig. 5b). These results indicate that the labeled DCs migrated into lymph nodes from the injection site over several days after the injection and gradually accumulated inside the lymph node.

Fluorescence images were obtained for the same individual mouse (Fig. 5c), which showed clear detection of the left popliteal lymph nodes by NIR fluorescence signals. These results were in accordance with *in vivo* MR imaging results, indicating that *in vivo* tracking of DCs via MR and fluorescence dual-mode imaging can be evaluated by labeling with ICGylated MNPs. Contrast-to-noise ratio (CNR) of each day indicates signal decrease in the lymph node over time (Fig. 5d), which was due to degradation of ICG molecules in the *in vivo* experiments.

It has been reported that the injection of many cells has negative effects on migration owing to a lack of nutrition at the localized site and the interruption of efficient flow along the lymphatic vessel^7, 43^. In that regard, we also performed DC migration assays using four times fewer cells, i.e., 5 × 10^5^ labeled DCs (Supplementary Fig. 7). As shown in Supplementary Fig. 7a, immediately after the injection of a small amount of DCs, darkening of lymph nodes was evident. The fluorescence of ICGylated MNPs after 1 day was much stronger than that after 2 or 3 days and thus the images cannot be shown at the same intensity scale. Fluorescence signals from the lymph nodes were observed for 3 days. Remarkably, even after 3 days after injection, fluorescence signals could be detected at the lymph node.

For clear observation of ICGylated MNPs by histological analysis, the popliteal lymph nodes of mice were extracted 3 days post-injection. Supplementary Fig. 8 shows NIR fluorescence and PB-stained images of the dissected and sliced lymph node. Co-localization of PB-stained and fluorescent regions was clearly identified for the case of the labeled DCs, suggesting the maintenance of the dual-mode structures and simultaneous sensitive detection, even at the tissue level.

It is difficult to use organic fluorescent dyes as imaging agents for long-term *in vivo* tracking owing to their rapid degradation and consequent low stabilities *in vivo*. A crucial point to note about this study is that the direct combination of MR and NIR fluorescence imaging agents (MNPs and ICG, respectively) are applicable for simultaneous, long-term *in vivo* dual-mode imaging without chemical modifications, expanding the potential clinical applications.

In conclusion, we designed a novel MR/NIR fluorescence imaging contrast agent by facile fluorescent dye-coating on MNPs. The fluorescent dye, ICG, was directly attached to the nanoparticle surface, enabling the nanoparticles to transition from a hydrophobic to a hydrophilic phase, a technique we termed “ICGylation”. ICG molecules were stably attached to MNPs. ICGylated MNPs and DCs labeled with ICGylated MNPs presented MR/NIR dual-mode imaging properties with high and sensitive detection. Importantly, the migration of the labeled DCs via lymphatic drainage and homing into lymph nodes was monitored by real-time MR/NIR imaging for three days. We expect that this novel MR/NIR contrast agent with sensitive detection and simultaneous imaging capabilities can be used in various fields, such as the imaging and tracking of immune cells to confirm immunotherapeutic efficacy. Furthermore, this principle can be also applied to the phase transfer of other kinds of hydrophobic particles using amphiphilic fluorescent dyes to develop multimodal probes for biomedical applications.

## Methods

### Synthesis of Magnetic Nanoparticles

Magnetic nanoparticles (MNPs, Fe_3_O_4_) were synthesized using previously reported methods^37^. Briefly, 36 g of Fe-oleate (40 mmol), 5.7 g of oleic acid (20 mmol), and 200 g of 1-octadecene were mixed under vacuum for 30 min at 25 °C. The mixture was heated to 320 °C at a constant heating rate of 3.3 °C/min and maintained at that temperature for 30 min. After cooling to room temperature, the products were precipitated by the addition of excess ethanol and isolated by centrifugation. The purified MNPs were dispersed in hexane with a concentration of 10 mg mL^−1^.

### Synthesis of ICGylated MNPs

MNPs in hexane were transferred to chloroform by adding excess methanol and centrifugation. MNPs (1 mg mL^−1^) in chloroform were filtered using a 0.1-µm syringe filter. Five milligrams of ICG was dissolved in water, which was added to an aqueous solution of ICG and emulsified using a microtip-probe sonicator (VCX750, 750 W, 20 kHz; SONICS, Newtown, CT, USA) set at 20% power for 3 min. The organic solvent was evaporated by magnetic stirring until it was completely removed. The uncoated ICG molecules were removed by magnetic separation several times at 4 °C.

### Characterization of ICGylated MNPs

The MNPs in organic phase and the ICGylated MNPs were characterized by HRTEM (Tecnai F20; Philips Electron Optics, Eindhoven, Holland) and their size distributions and surface zeta potentials were analyzed using the Zetasizer Nano ZS instrument (Malvern Instruments, Malvern, UK). An elemental analysis of MNPs was performed by inductively coupled plasma (ICP) optical emission spectroscopy (Optima 8300DV or 4300DV; Perkin Elmer, Waltham, MA, USA). The absorption and fluorescence emission spectra were obtained using a UV-Vis spectrophotometer (UV-2600; Shimadzu, Tokyo, Japan) and a fluorescence spectrophotometer (FS2; Scinco, Seoul, Korea), respectively. Powder forms of ICG, ICGylated MNPs, and MNPs were analyzed by FT-IR spectroscopy (ALPHA-P, Bruker, Bremen, Germany).

### Single-particle imaging of ICGylated MNPs

For single-particle imaging of the ICGylated MNPs, the solution was spin-coated on a cover slip, which was laid on an inverted microscope (IX71; Olympus, Tokyo, Japan). Dark-field and NIR fluorescence images were obtained using an electron multiplying charge coupled device (EMCCD) camera (DU-888E-C00-#BV; Andor Technology, Belfast, UK) *in situ*. For NIR fluorescence images, the cover slips were excited using a 785 nm continuous-wave (CW) diode-pumped solid state laser (PI-ECL-785-900-FC; Process Instruments, Salt Lake City, UT, USA), and BLP01-785R and FF01-842/SP (Semrock, Rochester, NY, USA) were used. For ROI analysis, the software Solis (Andor Technology) was used. To obtain color images of tissues, an sCMOS camera (OS4MPc-CL-RGB; Raptor Photonics, Larne, UK) was employed.

### Preparation of mouse bone marrow-derived dendritic cells (BMDCs)

Male C57BL/6 mice 5 to 6 weeks of age were purchased from OrientBio (Kyunggi-do, Korea). All experiments followed the guidelines of the committee on animal research at the Korea Basic Science Institute (KBSI), and the protocol was approved by the local institutional review committee on animal care (KBSI-AEC1508). Femurs and tibiae were collected, and the muscles were removed. Bones were washed with phosphate-buffered saline (PBS), their ends were cut with scissors, and the marrow was flushed out with RPMI 1640 media using a syringe (26 gauge needle). After they were washed in media, bone marrow cells were collected and cultured in dishes containing 10 mL of Roswell Park Memorial Institute (RPMI) medium supplemented with 10% fetal bovine serum, 50 IU/mL penicillin, 50 μg/mL streptomycin, and 20 ng/mL mouse recombinant granulocyte-macrophage colony-stimulating factor (GM-CSF; R&D Systems, Minneapolis, MN, USA). After 7 days, cells were harvested, washed, and used for *in vitro* and *in vivo* experiments^44^.

### *In vitro* and *in vivo* MR imaging studies

DCs were incubated with ICGylated MNPs at given concentrations for 24 h. After incubation, various quantities of cells were collected and mixed with agarose gel for the MR analysis. MR imaging was performed under a Bruker 4.7 T scanner (Biospec 47/40; Bruker). The MR images of phantoms were obtained by multi-slice multi-echo (MSME; TR = 3,600 ms, TE = 35 ms, slice thickness = 1 mm, FOV = 40 × 70 mm^2^, matrix = 192 × 192, NEX = 2) sequences. BMDCs were incubated with the ICGylated MNPs for 24 h and stimulated to maturation by lipopolysaccharide (LPS) addition. The cells labeled with ICGylated MNPs or unlabeled cells (2 × 10^6^ or 5 × 10^5^) were injected into TNF-α-pretreated (24 h before injection) mouse footpads. Prior to the imaging experiments, the mice were anesthetized with 4% isoflurane/O_2_ (v/v) and were maintained in a 1–2% (v/v) isoflurane/O_2_ atmosphere throughout the experiments. Axial images of the mouse popliteal or axillary lymph nodes were acquired using turbo-rapid acquisition with relaxation enhancement (Turbo-RARE) sequences using the following parameters: TR = 3,500 ms, TE = 36 ms, FOV = 30 × 30 mm^2^, slice thickness = 1 mm, matrix = 256 × 256, NEX = 4.

### *In vivo* fluorescence imaging studies

The DCs labeled with ICGylated MNPs (2 × 10^6^) were injected into the mouse footpads, and the imaging areas were treated with a depilatory cream. The mice were placed in an in-house-prepared high-quality *in vivo* imaging system connected to an EMCCD camera (DU897-EX, iXon Ultra; Andor Technology). Fluorescence images of the mice were acquired using an 808-nm (0–15 W) diode laser (OCLA, Gyeonggi-do, Korea) as an excitation light source and an emission filter (840 ± 12 nm, Semrock).

### Histological analysis

The dissected lymph nodes were embedded in a Tissue-Tek OCT compound (Sakura, Tokyo, Japan) and frozen in a deep freezer. Cryosections (~10 µm) were prepared using a cryostatic microtome (Leica Microsystems Nussloch GmbH; Nussloch, Germany) and transferred to glass slides. The slides were incubated with Prussian blue reagent for 15 min and the nuclei were counterstained with a pararosaniline solution for 30 s.

## Electronic supplementary material

Supplementary Information

## References

[1] Laurent S (2008). Magnetic iron oxide nanoparticles: Synthesis, stabilization, vectorization, physicochemical characterizations, and biological applications. Chem. Rev..

[2] Corot C, Robert P, Idée J-M, Port M (2006). Recent advances in iron oxide nanocrystal technology for medical imaging. Adv. Drug Deliv. Rev..

[3] Shubayev VI, Pisanic TR, Jin S (2009). Magnetic nanoparticles for theragnostics. Adv. Drug Deliv. Rev..

[4] Abu-Reziq R, Alper H, Wang D, Post ML (2006). Metal supported on dendronized magnetic nanoparticles: Highly selective hydroformylation catalysts. J. Am. Chem. Soc..

[5] Sun S, Murray CB, Weller D, Folks L, Moser A (2000). Monodisperse FePt Nanoparticles and Ferromagnetic FePt Nanocrystal Superlattices. Science.

[6] Ahrens ET, Bulte JWM (2013). Tracking immune cells *in vivo* using magnetic resonance imaging. Nat. Rev. Immunol..

[7] Wu C (2015). Negatively charged magnetite nanoparticle clusters as efficient MRI probes for dendritic cell labeling and *in vivo* tracking. Adv. Funct. Mater..

[8] Adams C (2016). Development of multifunctional magnetic nanoparticles for genetic engineering and tracking of neural stem cells. Adv. Healthc. Mater..

[9] Zhou S (2017). Labeling adipose derived stem cell sheet by ultrasmall super-paramagnetic Fe_3_O_4_ nanoparticles and magnetic resonance tracking *in vivo*. Sci, Rep..

[10] Lu A-H, Salabas EL, Schüth F (2007). Magnetic nanoparticles: Synthesis, protection, functionalization, and application. Angew. Chem. Int. Edit..

[11] Palma SICJ (2015). Effects of phase transfer ligands on monodisperse iron oxide magnetic nanoparticles. J. Colloid Interf. Sci..

[12] Khandhar AP, Ferguson RM, Simon JA, Krishnan KM (2012). Tailored magnetic nanoparticles for optimizing magnetic fluid hyperthermia. J. Biomed. Mater. Res. A.

[13] Liu Y (2014). Facile surface functionalization of hydrophobic magnetic nanoparticles. J. Am. Chem. Soc..

[14] Karakoti AS, Das S, Thevuthasan S, Seal S (2011). PEGylated inorganic nanoparticles. Angew. Chem. Int. Edit..

[15] Otsuka H, Nagasaki Y, Kataoka K (2003). PEGylated nanoparticles for biological and pharmaceutical applications. Adv. Drug Deliv. Rev..

[16] Xie J, Xu C, Kohler N, Hou Y, Sun S (2007). Controlled PEGylation of monodisperse fe_3_o_4_ nanoparticles for reduced non-specific uptake by macrophage cells. Adv. Mater..

[17] Tassa C, Shaw SY, Weissleder R (2011). Dextran-coated iron oxide nanoparticles: A versatile platform for targeted molecular imaging, molecular diagnostics, and therapy. Acc. Chem. Res..

[18] Yoon T-J (2006). Specific targeting, cell sorting, and bioimaging with smart magnetic silica core-shell nanomaterials. Small.

[19] Singh RK, Kim T-H, Patel KD, Knowles JC, Kim H-W (2012). Biocompatible magnetite nanoparticles with varying silica-coating layer for use in biomedicine: physicochemical and magnetic properties, and cellular compatibility. J. Biomed. Mater. Res. A.

[20] Demirer GS, Okur AC, Kizilel S (2015). Synthesis and design of biologically inspired biocompatible iron oxide nanoparticles for biomedical applications. J. Mater. Chem. B.

[21] Kharisov BI (2014). Solubilization, dispersion and stabilization of magnetic nanoparticles in water and non-aqueous solvents: Recent trends. RSC Adv..

[22] Wu W, He Q, Jiang C (2008). Magnetic iron oxide nanoparticles: synthesis and surface functionalization strategies. Nanoscale Res. Lett..

[23] Carney CE (2015). Nanodiscs as a modular platform for multimodal MR-optical imaging. Bioconjugate Chem..

[24] Tu C, Nagao R, Louie AY (2009). Multimodal magnetic-resonance/optical-imaging contrast agent sensitive to NADH. Angew. Chem. Int. Edit..

[25] Kim HM (2012). Self-fluorescence of chemically crosslinked MRI nanoprobes to enable multimodal imaging of therapeutic cells. Small.

[26] Chakravarty R (2014). Intrinsically germanium-69-labeled iron oxide nanoparticles: Synthesis and *in-vivo* dual-modality PET/MR imaging. Adv. Mater..

[27] Jaffer FA, Libby P, Weissleder R (2009). Optical and multimodality molecular imaging. Arterioscl. Throm. Vas..

[28] Hilderbrand SA, Weissleder R (2010). Near-infrared fluorescence: Application to *in vivo* molecular imaging. Curr. Opin. Chem. Biol..

[29] Sevick-Muraca EM, Houston JP, Gurfinkel M (2002). Fluorescence-enhanced, near infrared diagnostic imaging with contrast agents. Curr. Opin. Chem. Biol..

[30] Josephson L, Kircher MF, Mahmood U, Tang Y, Weissleder R (2002). Near-infrared fluorescent nanoparticles as combined MR/Optical imaging probes. Bioconjugate Chem..

[31] Marshall MV (2012). Near-infrared fluorescence imaging in humans with indocyanine green: A review and update. Open Surg. Oncol. J..

[32] Vinegoni C (2011). Indocyanine green enables near-infrared fluorescence imaging of lipid-rich, inflamed atherosclerotic plaques. Sci. Transl. Med..

[33] Schaafsma BE (2011). The clinical use of indocyanine green as a near-infrared fluorescent contrast agent for image-guided oncologic surgery. J. Surg. Oncol..

[34] Alander JT (2012). A review of indocyanine green fluorescent imaging in surgery. Int. J. Biomed. Imaging.

[35] Benson RC, Kues HA (1978). Fluorescence properties of indocyanine green as related to angiography. Phys. Med. Biol..

[36] Noh Y-W, Park HS, Sung M-H, Lim YT (2011). Enhancement of the photostability and retention time of indocyanine green in sentinel lymph node mapping by anionic polyelectrolytes. Biomaterials.

[37] Park J (2004). Ultra-large-scale syntheses of monodisperse nanocrystals. Nat. Mater..

[38] Desmettre T, Devoisselle JM, Mordon S (2000). Fluorescence properties and metabolic features of indocyanine green (ICG) as related to angiography. Surv. Ophthalmol..

[39] Hollins B, Noe B, Henderson JM (1987). Fluorometric determination of indocyanine green in plasma. Clin. Chem..

[40] Pisoni DS (2014). Symmetrical and asymmetrical cyanine dyes. synthesis, spectral properties, and BSA association study. J. Org. Chem..

[41] Muddana HS, Morgan TT, Adair JH, Butler PJ (2009). Photophysics of Cy3-encapsulated calcium phosphate nanoparticles. Nano Lett..

[42] Kohno Y (2014). Improved photostability of hydrophobic natural dye incorporated in organo-modified hydrotalcite. J. Phys. Chem. Solids.

[43] Moon H (2012). Noninvasive assessment of myocardial inflammation by cardiovascular magnetic resonance in a rat model of experimental autoimmune myocarditis. Circulation.

[44] Heo MB, Lim YT (2014). Programmed nanoparticles for combined immunomodulation, antigen presentation and tracking of immunotherapeutic cells. Biomaterials.

