# Supplementary material for: Convenient and effective ICGylation of magnetic nanoparticles for biomedical applications
Source: Sci Rep. 2017 Aug 18;7:8831. doi: 10.1038/s41598-017-09627-x (PMC5562755; doi:10.1038/s41598-017-09627-x)
Supplement: Supplementary file 1 — Supplementary Information [file 41598_2017_9627_MOESM1_ESM.doc]

**Supplementary Information**

Convenient and effective ICGylation of magnetic nanoparticles for biomedical applications

Hye Sun Park^1,2,★^, Jongwoo Kim^3,★^, Mi Young Cho^1,2^, Hyunseung Lee^1,2^, Sang Hwan Nam^3^, Yung Doug Suh^3,4^, & Kwan Soo Hong^1,2,5^

^1^Bioimaging Research Team, Korea Basic Science Institute, Cheongju 28119, Korea

^2^Immunotherapy Convergence Research Center, Korea Research Institute of Bioscience and Biotechnology, Daejeon 34141, Korea

^3^Laboratory for Advanced Molecular Probing (LAMP), Research Center for Convergence NanoRaman Technology, Korea Research Institute of Chemical Technology, Daejeon 34114, Korea

^4^School of Chemical Engineering, Sungkyunkwan University, Suwon 16419, Korea

^5^Graduate School of Analytical Science and Technology, Chungnam National University, Daejeon 34134, Korea

^★^These authors contributed equally to this work.

Correspondence and requests for materials should be addressed to K. S. H. (email: [*kshong@kbsi.re.kr*](mailto:kshong@kbsi.re.kr)) or Y. D. S. (email: [*ydsuh@krict.re.kr*](mailto:ydsuh@krict.re.kr)).


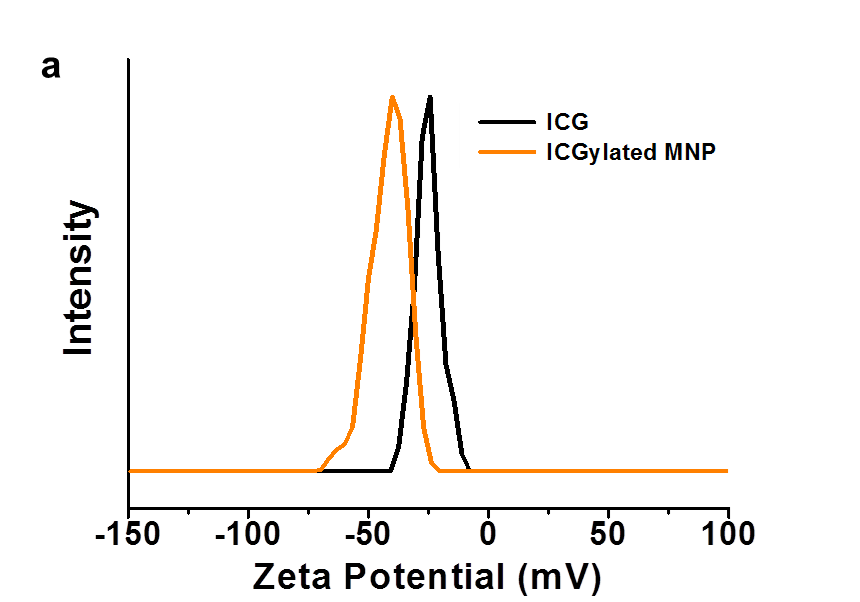


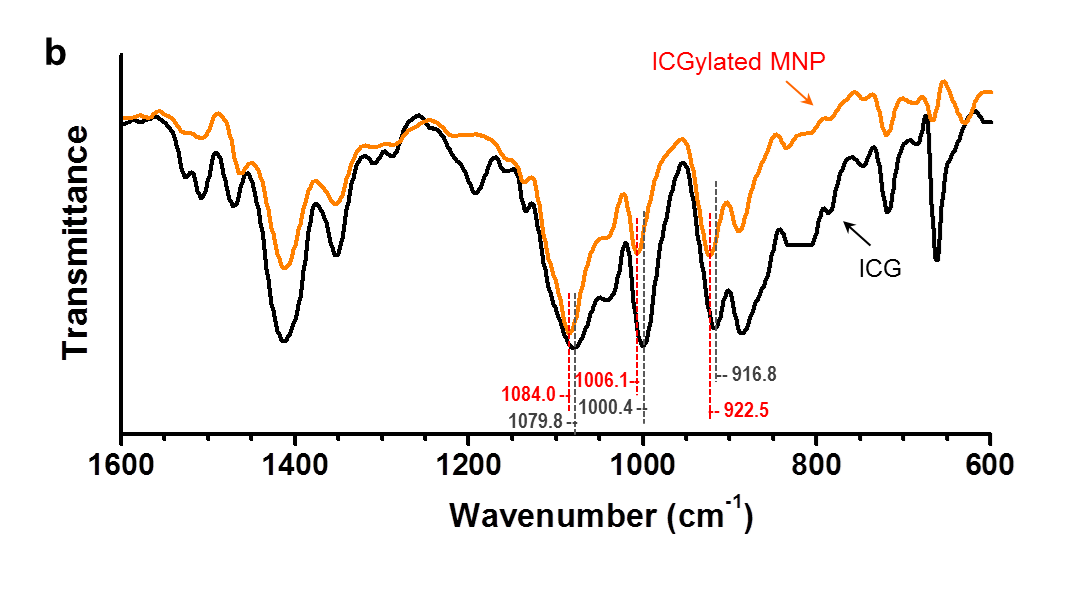


**Supplementary Figure 1.** a) Zeta potentials and b) FT-IR spectra of ICG and ICGylated MNPs.


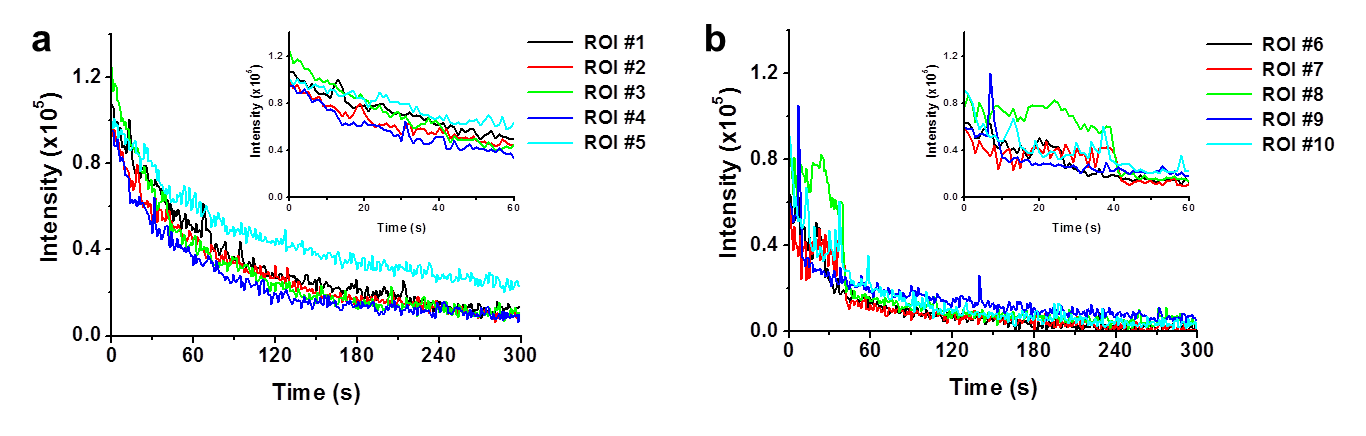


**Supplementary Figure 2.** Fluorescence intensities for a) ICGylated MNPs and b) uncoated (free) ICG under continuous irradiation at 785 nm. The inset shows the temporal behavior at early times. Each ROI is represented by a single particle image in Fig. 2.

**Supplementary Figure 3.** a) Flow cytometric analysis, b) *in vitro* images, and c) Prussian blue staining of DC2.4 cells labeled with ICGylated MNPs. d) Flow cytometric analysis, e) *in vitro* images, and f) Prussian blue staining of RAW264.7 cells labeled with ICGylated MNPs. The scale bars in b) and e) indicate 15 μm.

**Supplementary Figure 4.** Cell viability of BMDC, DC2.4, and RAW264.7 cells labeled with ICGylated MNPs as a function of Fe concentration.

**Supplementary Figure 5.** *In vitro* fluorescent stability of ICGylated MNPs in DCs. a) NIR fluorescence images of ICG-labeled DCs and b) those of ICG-MNP-labeled DCs. The initial fluorescent intensities of the cells were equally adjusted. c) Signal-to-noise ratios of fluorescence images for a) and b). ^*^p<0.05

**Supplementary Figure 6.** a) *In vivo* T_2_-weighted MR images of mouse axillary lymph nodes at various times after the injection of the labeled (L, left) and unlabeled (R, right) DCs (2×10^6^ cells). b) SNR values obtained from the MR images of a). c) *In vivo* NIR fluorescence images of mouse axillary lymph nodes after the injection of ICG-MNP-labeled DCs. d) CNR values of the lymph nodes obtained from c). ^**^p < 0.01.

**Supplementary Figure 7.** a) *In vivo* T_2_-weighted MR images of mouse popliteal lymph nodes at various time points after the injection of the labeled (L, left) and the unlabeled (R, right) DCs (5×10^5^ cells) and b) SNR values of the MR images. c) MR images and d) SNR values for axillary lymph nodes. ^**^p < 0.01, ^***^p < 0.001.

**Supplementary Figure 8.** a) *Ex vivo* NIR fluorescence images of the lymph nodes dissected after injection of the DCs labeled by ICGylated MNPs and unlabeled DCs. b) NIR fluorescence images and Prussian blue-stained images of the dissected lymph nodes after the injection of the labeled DCs and c) those of the unlabeled DCs.
